# Familial Occurrence of a Severe Phenotype of Central Serous Chorioretinopathy in Two Brothers

**DOI:** 10.7759/cureus.63557

**Published:** 2024-07-01

**Authors:** Yuta Inada, Yoichi Sakurada, Taiyo Shijo, Wataru Kikushima, Kenji Kashiwagi

**Affiliations:** 1 Department of Ophthalmology, University of Yamanashi, Chuo, JPN

**Keywords:** japanese sibling pair, choroidopathy, retina, inheritance, central serous chorioretinopathy

## Abstract

We report the familial occurrence of a severe phenotype of central serous chorioretinopathy (CSC). A 62-year-old man was referred to our institute to treat a macular lesion in his right eye. Best-corrected visual acuity (BCVA) in his right eye was 0.05 (decimal format). On the initial visit, swept-source optical coherence tomography (SS-OCT) demonstrated subretinal hyperreflective material (SHRM) and subretinal fluid involving the central macula in the right eye and a descending tract on fundus autofluorescence (FAF) in the left eye, and fluorescein angiography revealed focal leakage corresponding to choroidal vascular hyperpermeability (CVH) on indocyanine green angiography (ICGA) of the right eye. He received photodynamic therapy (PDT) for the right eye and exudation disappeared. His 66-year-old elder brother had a medical history of CSC in both eyes and had received treatment at our hospital at 61 years old. On the initial presentation, ICGA showed multiple CVH in both eyes, and FAF showed hypofluorescence corresponding to retinal pigment epithelium (RPE) tears and RPE atrophy in both eyes. Bullous retinal detachment (RD) developed inferiorly in both eyes, and a vitrectomy was performed for the right eye to repair RD. The baseline BCVA was 0.3 in both eyes. Two years after the initial visit, recurrent serous RD developed in his left eye, and multiple PDT sessions were performed during the six-year follow-up. A severe phenotype of CSC may be associated with a genetic background.

## Introduction

Central serous chorioretinopathy (CSC) is a pachychoroid spectrum disorder characterized by serous retinal detachment (RD) with or without serous detachment of the retinal pigment epithelium (RPE) in the posterior pole [1]. Most of the eyes with CSC show CVH and increased choroidal thickness on indocyanine green angiography and spectral-domain/swept-source optical coherence tomography (SD/SS-OCT), respectively. Recently, genome-wide association studies have revealed several single nucleotide polymorphisms associated with CSC [2-5]. Although its pathogenesis is not fully understood, the choroid is considered one of the major regions involved in CSC etiology [1].

To date, few studies have described the familial occurrence of CSC [6,7]. A previous study examining relatives of patients with characteristic CSC showed that 35 (44%) of 80 relatives had fundus lesions, including 22 cases with chronic CSC and 13 cases with RPE atrophy [6]. These results indicate the presence of a genetic predisposition associated with CSC. The clinical presentation of CSC varies depending on age, sex, and genetic factors [8-10]. However, to date, reports demonstrating a severe phenotype of CSC in family members are rare.

In this case report, we describe the familial occurrence of a severe phenotype of CSC in siblings.

## Case presentation

Case 1 and Case 2 presented to the University of Yamanashi in November 2022 and July 2015, respectively. These cases constitute a consecutive series involving brothers.

Case 1

A 62-year-old male was initially referred to the Yamanashi University Hospital due to visual deterioration in the right eye. The best-corrected visual acuity (BCVA) was 0.05 in the right eye and 1.0 in the left eye, both in a decimal format. The sensory retinal thickness was 98 and 166 μm in the right and left eyes, respectively. The outer nuclear thickness was 58 and 98 μm in the right and left eyes, respectively. The axial length was 23.17 and 23.27 mm in the right and left eyes, respectively. He did not have a history of taking steroids.

**Figure 1:**
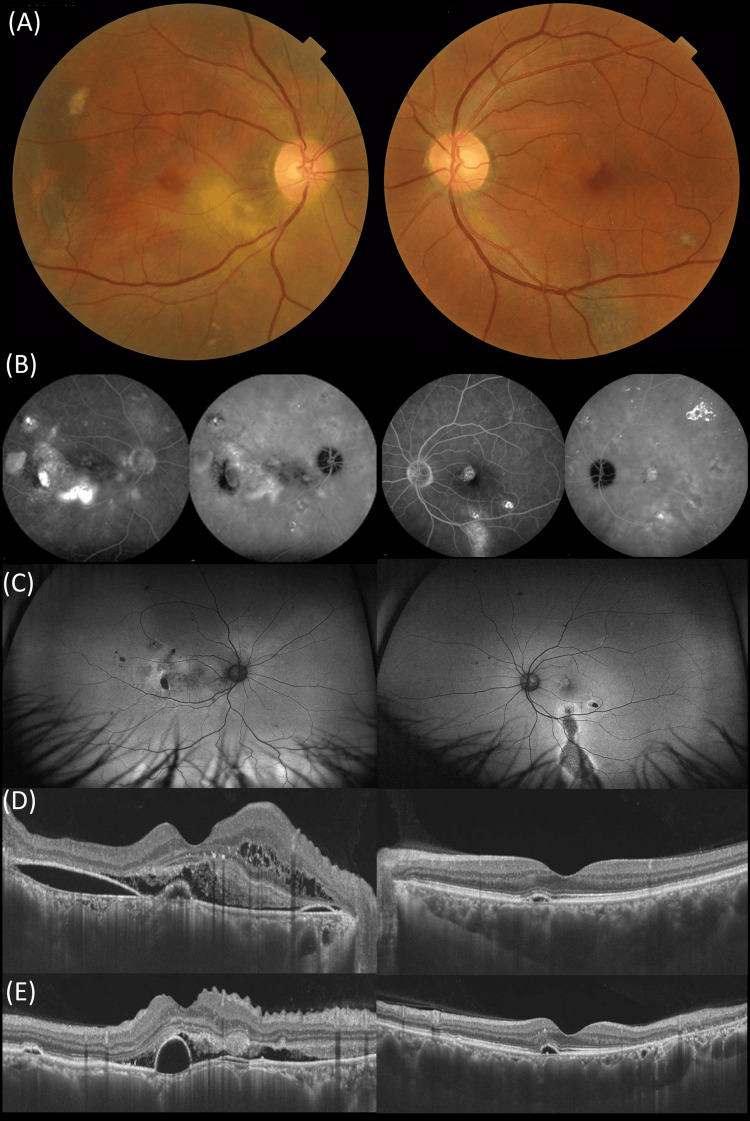
Multimodal imaging of a 62-year-old male (Case 1) presenting with central serous chorioretinopathy on the initial presentation (A) Color fundus photography showed a yellowish lesion in the macula and RPE abnormality inferior to the macula in the right and left eyes, respectively. (B) A fluorescein angiography showed diffuse leakage inferior to the macula corresponding to CVH on ICGA and a pinpoint hyperfluorescent superior to the temporal macula in the right eye. Indocyanine green showed diffuse CVH in both eyes. (C) A wide-field fundus autofluorescence denoted a few hypoautofluorescence corresponding to RPE abnormalities and a descending tract in the right and left eyes, respectively. A horizontal (D) and vertical (E) SS-OCT demonstrated subretinal hyperreflective material and small RPE detachment in the right and left eyes, respectively. RPE: Retinal pigment epithelium; CVH: Choroidal vascular hyperpermeability; ICGA: Indocyanine green angiography; SS-OCT: Swept-source optical coherence tomography.

At the initial visit, a comprehensive examination was conducted, including color fundus photography, SS-OCT, and fluorescein and indocyanine green angiography (FA/ICGA) using Spectralis (HRA2, Heidelberg Engineering, Dossenheim, Germany) (Figure 1). Color fundus photography revealed yellowish-white lesions around the macula in the right eye and RPE depigmentation inferiorly in the macula of the left eye. A horizontal SS-OCT scan demonstrated a large amount of fibrin and subretinal fluid in the right eye as well as RPE and outer retinal atrophy inferiorly in the macula of the left eye. The subfoveal choroidal thicknesses were 790 (right eye) and 550 μm (left eye), respectively, and the central retinal thicknesses were 385 (right eye) and 179 μm (left eye), respectively. Ultra-wide field fundus autofluorescence showed hypoautofluorescence sparing the fovea, indicating chronic CSC in the left eye. Late-phase wide-field FA showed staining corresponding to that in late-phase ICGA in the right eye.

**Figure 2:**
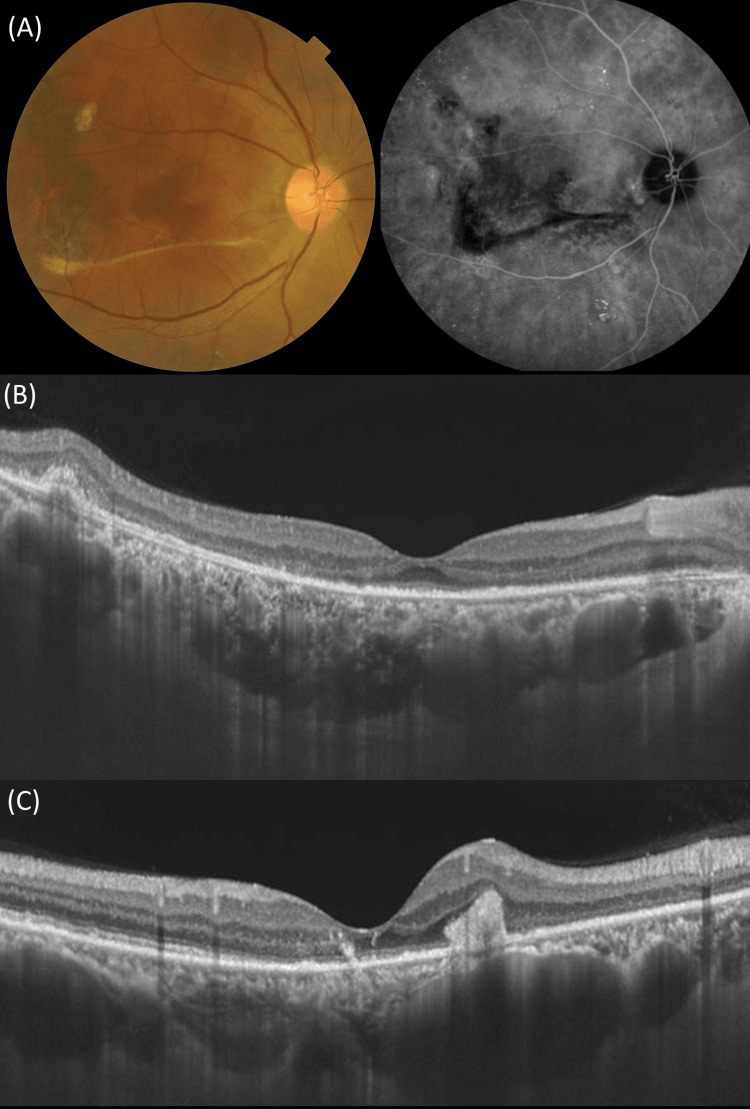
Multimodal imaging of the right eye (Case 1) three months after photodynamic therapy (A) Color fundus photography demonstrates the disappearance of yellowish materials but shows a retinal fold resembling a strand (left). Indocyanine green angiography shows the disappearance of choroidal vascular hyperpermeability. (B, C) Swept-source optical coherence tomography demonstrates a decrease in the central choroidal thickness (485 μm) and retinal thickness (145 μm) in a horizontal scan (B) through the fovea and the presence of hyperreflective material in a vertical scan (C) through the fovea.

Based on multimodal imaging, the right and left eyes were diagnosed with CSC with fibrinous tissue and inactive chronic CSC with a descending tract, respectively. Five days after the initial visit, full doses of photodynamic therapy (PDT) were administered to the right eye as previously described [11]. The greatest linear dimension and spot size were 4100 and 5100 μm, respectively. Three months after PDT, residual fibrinous tissue and subretinal fluid completely disappeared on SS-OCT, and BCVA improved from 0.05 to 0.3 in the right eye (Figure 2).

Case 2

This patient had an older sister and brother. The older sister died in her 30s due to a brain tumor. Seven years before the initial visit, the 66-year-old brother had received treatment for bullous CSC in his right eye. Similar to Case 1, Case 2 did not have a history of taking steroids. Bullous CSC was diagnosed by multimodal imaging, including ultra-widefield color fundus photography, FAF, and ICGA. In addition to bullous RD without retinal tears, FAF showed RPE tears, as often seen in bullous CSC, and ICGA showed multiple CVH. He was 61 years old at the initial presentation, and BCVA was 0.3 in both eyes. The sensory retina thicknesses were 89 and 92 μm in the right and left eyes, respectively. Outer nuclear thicknesses were 58 and 61 μm in the right and left eyes, respectively. The axial length of Case 2 was 23.57 and 23.46 mm in the right and left eyes, respectively.

**Figure 3:**
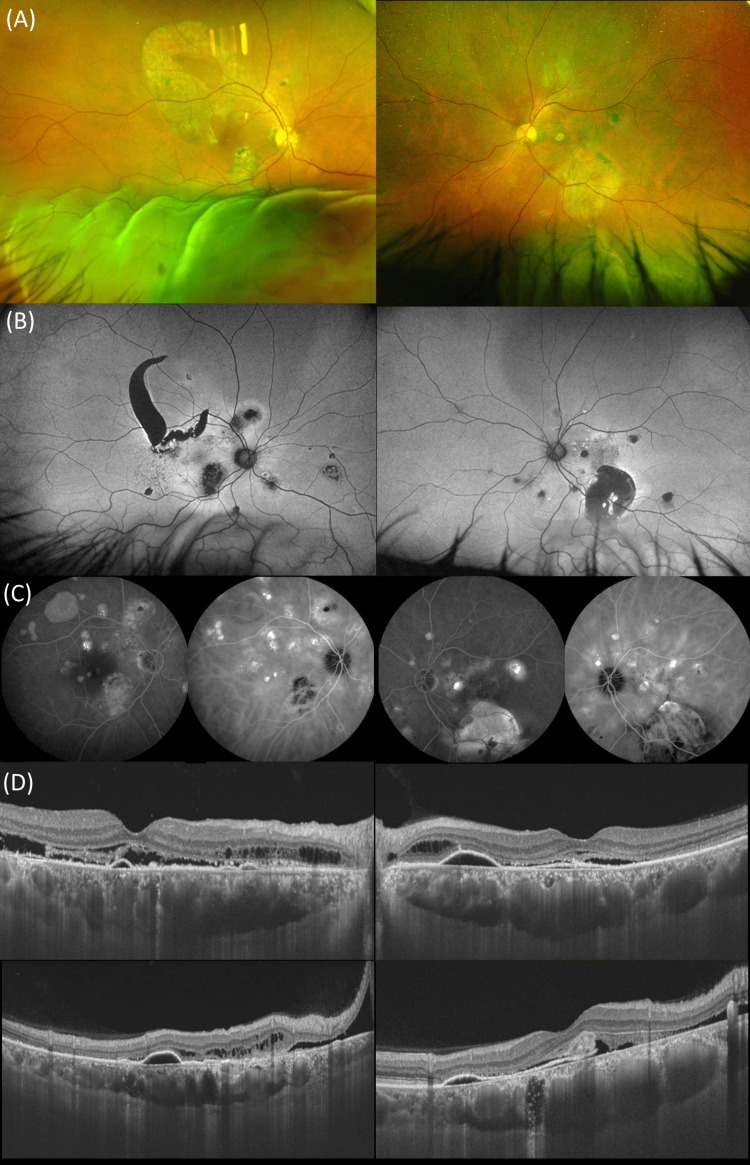
Multimodal imaging of a 62-year-old elder brother (Case 2) with bullous CSC in both eyes (A) Wide-field color fundus photography shows the presence of bullous retinal detachment inferiorly in both eyes. (B) Wide-field fundus autofluorescence demonstrates the presence of hypoautofluorescence corresponding to RPE atrophy in both eyes and large RPE tears in the right eye. (C) Indocyanine green angiography shows multiple cyanescence corresponding to choroidal vascular hyperpermeability in both eyes. (D) Swept-source optical coherence tomography demonstrates shallow serous detachment in both eyes. RPE: Retinal pigment epithelium.

Figure 3 shows multimodal imaging on the initial visit. ICGA shows multiple CVH and a wide-field FAF shows hypofluorescence corresponding to RPE atrophy in both eyes and large RPE tears in the right eye. To repair the bullous RD without retinal holes/tears, he initially underwent a vitrectomy with SF6 gas tamponade for the right eye with bullous RD. Subsequently, PDT was applied to the right eye with residual subretinal fluid presumed from the choroid. In addition, PDT was applied to the left eye with subretinal fluid secondary to CSC. At the last visit (February 2023), seven years after the initial treatment, BCVA was 0.1 and 0.5 in the right and left eyes, respectively.

Written consent was obtained from the patients for the publication of case details and images.

## Discussion

Classically, CSC is classified into two types, acute and chronic, depending on the duration of the disease. However, this classification mostly relies on patient subjectivity, and patients with foveal-sparing CSC rarely notice the onset of the disease. Recently, the CSC International Group classified CSC into three types: simple, complex, and atypical [12]. Depending on the presence or absence of the RPE alteration area, CSC is subclassified into simple or complex CSC, and the bullous CSC was defined as atypical CSC. In this case report, we present a severe phenotype of CSC in two brothers; the elder brother represents an atypical type with bullous and exudative RD, while the younger brother represents a complex type.

Although several variants susceptible to CSC have been identified, their mode of inheritance has not yet been determined. However, a previous report demonstrated that pachychoroid could be a dominantly inherited condition [13]. In the present study, the subfoveal choroidal thickness of the younger brother was 790 and 550 μm in the right and left eyes, respectively, while that of the elder brother was 400 and 500 μm in the right and left eyes, respectively. Considering age and sex, the subfoveal choroidal thickness of the two cases was greater than that of previously reported 249 Japanese male CSC patients (mean subfoveal choroidal thickness: 402 ± 115, mean age: 55.1 ± 11.3 years) [9]. A recent genome-wide association study reported that increased subfoveal choroidal thickness was associated with variants of the CFH gene in normal subjects, and several genome-wide association studies demonstrated that CFH variants were associated with CSC [2,4]. We recently reported that complex CSC had significantly more risk alleles of the CFH gene than simple CSC [10]. Taken together, CFH might not only be associated with CSC development but also with disease severity.

In systemic diseases other than eyes, bilateral involvement implicates a stronger genetic background compared with unilateral involvement [14,15]. Further, in eyes with polypoidal choroidal vasculopathy, patients with bilateral involvement can have a more severe phenotype compared to those with unilateral involvement [16]. Recently, it was reported that bilateral involvement is significantly higher in complex CSC compared with simple CSC, along with a higher risk variant of the CFH gene [10]. In the present report, both cases demonstrated bilateral involvement, which may indicate a severe phenotype due to a stronger genetic background. In the daily clinic, physicians need to take a family history for patients with CSC. In the future, genetic testing might be prevalent to predict a prognosis for CSC patients. Bullous CSC is a rare phenotype in CSC, and RPE tears and fibrinous tissues are common findings [17]. In the present study, both patients exhibited subretinal hyperreflective materials similar to fibrin on SS-OCT.

Although the younger brother did not develop bullous RD, the manifestation was similar between the brothers, including bilateral involvement and the development of SHRM. Although we cannot draw a definitive conclusion about these brothers, it would be interesting to investigate the association between the manifestation and family history using more cohorts with family history. In both cases, the patients never used steroids and never smoked. There is a high possibility that they share most lifestyle habits. Besides a genetic background, environmental factors might have influenced the onset of this condition. In addition, there is a possibility that similar lifestyle habits led to similar manifestations of CSC.

Limitations of the study

We did not perform optical coherence tomography angiography (OCTA) for either case. Therefore, there is a possibility that macular neovascularization developed in one or both cases. A recent study found that pretreatment BCVA and thickness from the internal limiting membrane and external limiting membrane were better predictors of PDT in eyes with CSC [18]. Further studies are needed to determine if these predictors are applicable to treatments other than PDT.

In Case 1, PDT was performed on the right eye. Although a complete resolution was achieved with one session of PDT, a recent study demonstrated that complex CSC required additional treatments than simple CSC [19], suggesting that this case might also require multiple treatments in the long-term follow-up.

## Conclusions

When patients present with CSC, taking a family history might be useful for predicting unilateral or bilateral involvement of the eyes and the long-term visual prognosis. This case report is one of the few demonstrating the severe phenotype of CSC in siblings. To confirm the heritability of CSC severity, it is necessary to investigate a large number of families with CSC.
